# 266. Nasal Microbiome and the Effect of Nasal Decolonization with a Novel PovidoneIodine Antiseptic Solution: A Prospective and Randomized Clinical Trial

**DOI:** 10.1093/ofid/ofad500.338

**Published:** 2023-11-27

**Authors:** Diana Fernández-Rodríguez, Jeongeun Cho, Emanuele Chisari, Javad Parvizi

**Affiliations:** MD/PhD Plan de Estudios Combinados en Medicina (PECEM), Mexico City, Distrito Federal, Mexico; Rothman Orthopaedic Institute, Philadelphia, Pennsylvania; Rothman Orthopaedic Institute, Philadelphia, Pennsylvania; Rothman Orthopaedic Institute, Philadelphia, Pennsylvania

## Abstract

**Background:**

The presence of pathogenic bacteria in the anterior nares has gained substantial attention due to its association with an increased risk for surgical site infections. Nasal decolonization has been shown to reduce the risk for SSIs. Recent studies have suggested that the profile of the nose microbiome is a determining factor for the susceptibility to develop SSIs. Therefore, this prospective clinical trial was set up to study the profile of nasal microbiome and test the effect of a specific nasal decolonization solution on the microbiome.

**Methods:**

We conducted a prospective study on 50 individuals who were randomly assigned to receive a nasal antiseptic solution (n=25) or a control PBS solution (n=25). The antiseptic solution (containing povidone-iodine [PVP-I] as the main ingredient) or PBS was administered with a cotton swab and a rotatory movement for 15 s in the anterior part of both nares. Nasal swabs were obtained before application (baseline) and at 3 different timepoints after application (5 min, 2 h, and 24 h). Nasal swabs were subjected next generation sequencing (NGS) analyses and cultured in trypticase soy agar plates.

Study design

**Figure:**
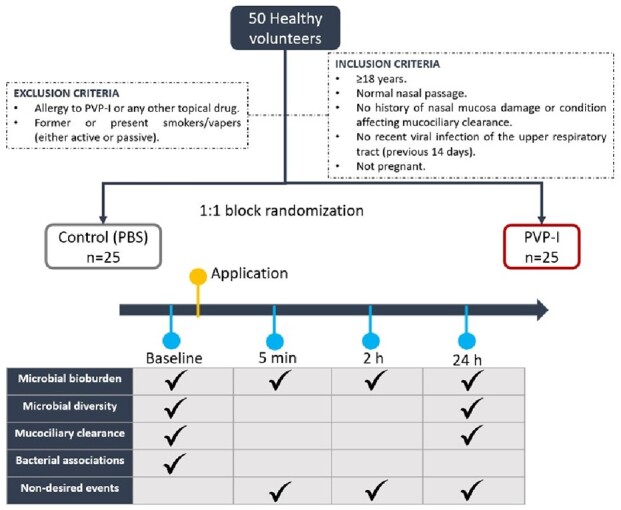

Fifty volunteers were recruited for this randomized clinical trial. Block randomization was performed to determine group allocation, thus, PVP-I (n=25) or PBS (n=25) application in the anterior nares. We assessed bioburden at baseline, 5 minutes, 2 hours, and 24 hours post-application, meanwhile microbial diversity and mucociliary clearance was assessed at baseline and 24 hours post-application. Bacterial associations were assessed at baseline and non-desired events were examined at every follow-up visit.

**Results:**

A high bioburden reduction was observed after the application of PVP-I (log_10_ 3.538 ± 0.918 at 5 min; log_10_ 2.961 ± 1.466 at 2 hours; and log_10_ 0.849 ± 1.092 at 24 hours; p< 0.01), compared to PBS. At baseline, there were substantial associations between anaerobic species, *Corynebacterium* spp, *Staphylococcus* spp, and *Dolosigranulum* spp. The top species affected by the treatment were *C. acnes, Staphylococcus,* and *Corynebacterium* species, among others. None of the subjects in the study experienced any adverse effects associated with this study, nor increases in MCC time.

Bioburden reduction (A) and mucociliary clearance assessment (B)

**Figure:**
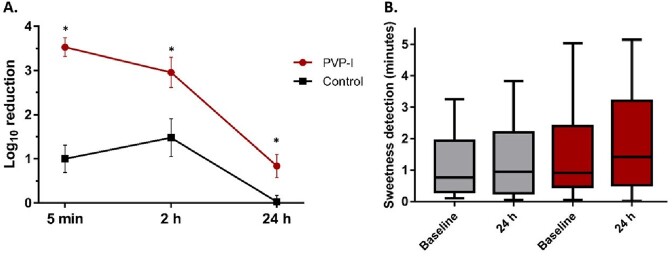

A) Data is expressed as mean ± SEM CFU log10 reduction, from baseline bioburden. B) Sweetness detection, in minutes, at baseline and 24 hours post-application.

Bacterial composition.

**Figure:**
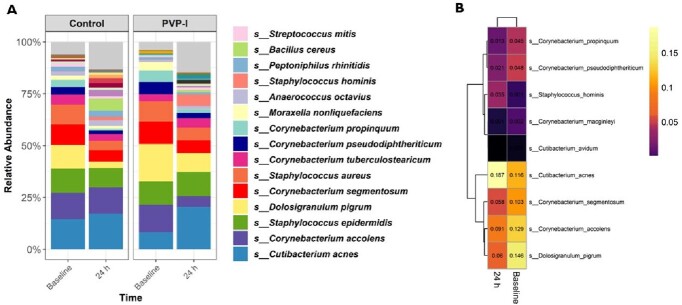

A) Relative abundance of the most prevalent bacterial species and B) heatmap illustrating the mean relative abundance of bacteria detected to be differentially abundant by ANCOMBC procedure. A cell colored in black can be considered “true” zero, whereas there were no detections of a particular bacteria for that given cell.

Network plot shows associations among bacterial species with p<0.10.

**Figure:**
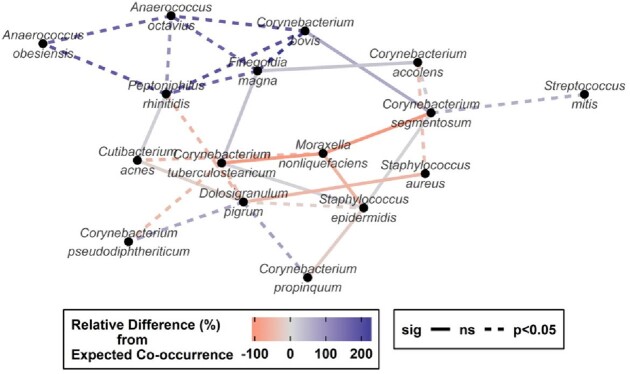

Each node represents a species and is linked by species associations.

**Conclusion:**

There was a considerable difference in the profile of nasal microbiome organisms between individuals. Intranasal application of the PVP-I antiseptic led to a substantial reduction of the nasal flora for up to 24 hours after application. Restoration of the microbial population was observed with slight variations to the baseline profiles. Antiseptic solutions applied to the anterior nares can transiently and markedly reduce the bioburden of the nose.

**Disclosures:**

**Javad Parvizi, MD, FRCS**, 3M: Grant/Research Support|Acumed, LLC: Stocks/Bonds|Aesculap: Grant/Research Support|Alphaeon: Stocks/Bonds|AO Spine: Stocks/Bonds|Becton Dickenson: Advisor/Consultant|Biomet: Grant/Research Support|Cardinal Health: Advisor/Consultant|Cempra: Grant/Research Support|CeramTec: Grant/Research Support|Ceribell: Stocks/Bonds|Coracoid: Stocks/Bonds|Corentec: Advisor/Consultant|Datatrace: Grant/Research Support|DePuy: Grant/Research Support|Elsevier: Grant/Research Support|Elute: Stocks/Bonds|Ethicon: Advisor/Consultant|Hip Innovation Technology: Stocks/Bonds|Illuminus: Stocks/Bonds|Integra: Grant/Research Support|Intellijoint: Stocks/Bonds|Jaypee Publishers: Grant/Research Support|KCI / 3M (Acelity): Advisor/Consultant|Lima: Grant/Research Support|MicroGenDx: Advisor/Consultant|Molecular Surface Technologies: Stocks/Bonds|Myoscience: Grant/Research Support|Nanooxygenic: Stocks/Bonds|National Institutes of Health (NIAMS & NICHD): Grant/Research Support|NDRI: Grant/Research Support|Novartis: Grant/Research Support|OREF: Grant/Research Support|Orthospace: Grant/Research Support|Osteal: Stocks/Bonds|Parvizi Surgical Innovations and Subsidiaries: Stocks/Bonds|Peptilogic: Stocks/Bonds|Peptilogics: Advisor/Consultant|Pfizer: Grant/Research Support|PRN-Veterinary: Grant/Research Support|Rotation Medical: Grant/Research Support|Simplify Medical: Grant/Research Support|SLACK Incorporated: Grant/Research Support|Smith & Nephew: Grant/Research Support|Sonata: Stocks/Bonds|Stelkast: Grant/Research Support|Stryker: Grant/Research Support|Synthes: Grant/Research Support|Tenor: Advisor/Consultant|TissueGene: Grant/Research Support|Tornier: Grant/Research Support|Wolters Kluwer Health - Lippincott Williams & Wilkins: Grant/Research Support|Zimmer Biomet: Advisor/Consultant|Zimmer Biomet: Grant/Research Support

